# Decoding Behcet’s Uveitis: an In-depth review of pathogenesis and therapeutic advances

**DOI:** 10.1186/s12974-024-03123-6

**Published:** 2024-05-22

**Authors:** Yuxuan Guan, Fuzhen Li, Na Li, Peizeng Yang

**Affiliations:** 1 Department of Ophthalmology, Henan International Joint Research Laboratory for Ocular Immunology and Retinal Injury Repair, The First Affiliated Hospital of Zhengzhou University, Henan Province Eye Hospital, Zhengzhou, 450052 People’s Republic of China; 2 The Academy of Medical Sciences, Zhengzhou University, Zhengzhou, 450052 People’s Republic of China

**Keywords:** Behcet’s disease, Behcet’s uveitis, Adaptive immunity, Innate immunity, Biologics

## Abstract

Behcet’s disease (BD) is a rare but globally distributed vasculitis that primarily affects populations in the Mediterranean and Asian regions. Behcet’s uveitis (BU) is a common manifestation of BD, occurring in over two-thirds of the patients. BU is characterized by bilateral, chronic, recurrent, non-granulomatous uveitis in association with complications such as retinal ischemia and atrophy, optic atrophy, macular ischemia, macular edema, and further neovascular complications (vitreous hemorrhage, neovascular glaucoma). Although the etiology and pathogenesis of BU remain unclear, numerous studies reveal that genetic factors (such as *HLA-B51*), dysregulated immune responses of both the innate and adaptive immune systems, infections (such as streptococcus), and environmental factors (such as GDP) are all involved in its development. Innate immunity, including hyperactivity of neutrophils and γδT cells and elevated NK1/NK2 ratios, has been shown to play an essential role in this disease. Adaptive immune system disturbance, including homeostatic perturbations, Th1, Th17 overaction, and Treg cell dysfunction, is thought to be involved in BU pathogenesis. Treatment of BU requires a tailored approach based on the location, severity of inflammation, and systemic manifestations. The therapy aims to achieve rapid inflammation suppression, preservation of vision, and prevention of recurrence. Systemic corticosteroids combined with other immunosuppressive agents have been widely used to treat BU, and beneficial effects are observed in most patients. Recently, biologics have been shown to be effective in treating refractory BU cases. Novel therapeutic targets for treating BU include the LCK gene, Th17/Treg balance, JAK pathway inhibition, and cytokines such as IL-17 and RORγt. This article summarizes the recent studies on BU, especially in terms of pathogenesis, diagnostic criteria and classification, auxiliary examination, and treatment options. A better understanding of the significance of microbiome composition, genetic basis, and persistent immune mechanisms, as well as advancements in identifying new biomarkers and implementing objective quantitative detection of BU, may greatly contribute to improving the adequate management of BU patients.

## Introduction

Behcet’s disease (BD), also known as Behcet’s syndrome [1], is a rare chronic recurrent vasculitis with unclear etiology and pathogenesis. Up to date, BD is considered a heterogeneous disease with close association with genetics (e.g., *HLA-B51*), immunity (innate and adaptive immunity), infections (e.g., streptococcus), and the environment (e.g., GDP) [2–6]. More than 60% of BD patients have eye lesions, which can be the primary or only manifestation of BD. The most common eye lesion is uveitis, typically manifesting as recurrent bilateral non-granulomatous uveitis. Behcet’s uveitis (BU) represents an immune-mediated intraocular inflammatory disorder with potential risk of blinding [7, 8]. The unique complications of BU are of high concerns, such as retinal ischemia and atrophy, optic atrophy, macular ischemia, macular edema, and further neovascular complications (vitreous hemorrhage, neovascular glaucoma). These are common complications that lead to permanent visual loss. It can develop alone or with systemic manifestations. Although modern immunosuppressive agents have improved BU prognosis, approximately 20.4% of eyes become blind due to recurrent episodes [8, 9].

In the therapeutic landscape of BU, the enduring pillars of glucocorticoids and immunosuppressants have been recently complemented by the emergence of biologics, providing new, promising management for this disease. The impetus for these advancements has been catalyzed by fast-paced strides in the fields of genetics, immunology, and technology, thus driving significant breakthroughs in both experimental and clinical research in BU.

Herein, this review article focuses on recent advances in understanding the immunologic etiology and therapeutic advances that contribute to the pathogenesis of BU. In addition to this, some insights are provided on how to improve the diagnosis and management of BU in practice.

### History

BD has its earliest recorded description in the third book of endemic diseases by Hippocrates. He indicated that the Mediterranean region and Asian populations were most affected by BD, which is how BD earned the name old Silk Road disease [10]. The disease was first recognized by Hulusi Behçet in 1937, and it is characterized by a triad of recurrent clinical symptoms: oral ulcers, genital ulcers, and ocular lesions [11, 12]. BD can be diagnosed when oral ulcers are present and at least two of the following criteria are met: distinctive ocular lesions, typical skin lesions, recurrent genital ulcers, or a positive skin pathology test [13, 14]. BD symptoms can be erratic, with symptomatic or remission periods lasting months, years, or decades. This condition has been given various names (Table 1).

**Table 1:** Nomenclature Variations of BD

Variation	Description
Behcet’s Syndrome or Behcet’s Disease	The most commonly used names worldwide, named after Hulusi Behçet who first described the disease.
Silk Road Disease	This older term, less commonly used today, reflects the high prevalence of the disease along the historical Silk Road.
Neuro-Behcet’s, Ocular-Behcet’s	These terms refer to cases where the disease primarily affects a specific system.
Adamantiades-Behcet’s disease	This name is commonly used in Greece and acknowledges the work of Benedictos Adamantiades.
Behcet’s-associated Uveitis (BDU) or Behcet’s uveitis (BU)	This refers to uveitis in BD.

### Diagnostic and classification criteria

In clinical practice, there are no specific diagnostic tests or histological features that can definitively identify BD. Current diagnostic criteria rely on clinical symptoms and imaging findings.

The International Study Group for Behcet’s Disease (ISG), established in 1990, is widely used as the first truly international standard with high specificity. Recurrent oral ulcers plus 2 other criteria, including recurrent genital ulcers, eye lesions, skin lesions, and positive pathological tests, are sufficient for diagnosis [14]. Recurrent oral ulcers are necessary for diagnosis, but the oral manifestations of patients in the early stages of the disease are not completely consistent. Moreover, the differences in the prevalence of diseases in different regions were not considered in the formulation of the criteria, so some regions with higher prevalence were ignored. In contrast, the International Criteria for Behcet’s Disease (ICBD), developed in 2014, incorporates neurological and vascular manifestations, improving diagnostic sensitivity but reducing specificity [15]. Although useful for diagnostic guidance, all criteria ignore the baseline probability of disease in patients and may be more beneficial for differential diagnosis in non-endemic areas. The Standardization of Uveitis Nomenclature (SUN) working group proposed in 2021 provides a unique framework for the identification of BU and other non-infectious uveitis. In particular, this classification standard includes focal retinal infiltration in the definition of ocular lesions, which improves diagnostic accuracy and is suitable for clinical and translational studies [16]. It is limited by specificity and may inadvertently exclude cases with atypical manifestations or overlapping with other uveitis entities. Therefore, clinicians should exercise caution when evaluating suspected BU patients and consider the broader clinical context. In addition, Tugal-Tutkun et al. ‘s algorithm offers a promising way to diagnose adult BU based solely on ocular manifestations, providing a solution to clinicians’ bedside challenges [17].

The absence of universally acknowledged scoring criteria for BD poses a challenge. However, the Ocular Behcet Disease Research Group of Japan introduced the Behcet’s disease ocular attack score 24 (BOS24) scoring system in 2014, which can evaluate the clinical inflammatory activity.

BOS24 serves as a comprehensive measure for assessing ocular inflammation. This scoring system encompasses six distinct parameters, all of which are evaluated on a per-ocular episode basis. The parameters encompass various aspects related to the eye, such as abnormalities in the vitreous, lesions located at the subfoveal area, lesions found in the peripheral region of the fundus, presence of cells in the anterior chamber, lesions affecting the posterior pole, and lesions affecting the optic disc. This particular system of classification divides the retinal field into two main areas: the posterior pole and the peripheral retina. The peripheral retina is then further divided into quadrants for a more detailed analysis and understanding. This innovative system incorporates a set of specific parameters that are carefully chosen to ensure consistency and accuracy in quantifying the severity of ocular inflammation. Through the implementation of these parameters, the BOS24 establishes a standardized approach to assess the level of ocular inflammation in BD patients [18].

Notably, BOS24 incorporates the grading scales developed by the SUN working group for scoring anterior chamber cells, and follows the system proposed by Nussenblatt et al. for evaluating vitreous opacity [19, 20].

An important attribute of BOS24 is its reliance solely on objective ocular findings per episode, explicitly excluding patient-reported symptoms or subjective examination outcomes such as visual acuity. Moreover, the scoring system focuses specifically on new inflammatory manifestations, excluding chronic inflammatory signs. This clear-cut delineation ensures that BOS24 accurately captures the acute inflammatory burden in each episode of BU.

In addition to diagnostic criteria, the differential diagnosis of BU is equally important (Table 2).

**Table 2:** Differential diagnosis for BU

Major clinical manifestation	Differential diagnoses
Anterior uveitis	Ankylosing spondylitis Inflammatory bowel disease Reactive arthritis (Reiter’s syndrome) HLA-B27-associated uveitis Psoriatic arthritis Herpetic anterior uveitis Idiopathic anterior uveitis
Posterior uveitis	Systemic lupus erythematosus-associated retinal vasculitis ANCA-associated vasculitis Cytomegalovirus retinitis Syphilitic retinitis Frosted branch angiitis Toxoplasmosis Eales disease (retinal venous perivasculitis) Acute retinal necrosis syndrome Masquerade syndrome Idiopathic retinal vasculitis
Panuveitis	Idiopathic panuveitis with occlusive vasculitis Sarcoidosis Multiple sclerosis Tubulointerstitial nephritis and uveitis syndrome Masquerade syndrome Tuberculous uveitis Fungal endophthalmitis Bacterial endophthalmitis

### Epidemiology

BU exhibits a distinct geographical distribution, primarily observed along the ancient Silk Road, extending across the vast expanse from East Asia to the Mediterranean region [21]. Turkey records the highest incidence rate, with an estimated 420 cases per 100,000 individuals [22]. Noteworthy prevalence is also observed in Iran, Korea, Japan, Greece, Israel, and Saudi Arabia [15]. Although the incidence in North America and Europe is lower, cases have been identified on all continents [23]. This distribution pattern suggests a rational association with genetic factors, potentially linked to the *HLA-B51* gene [24].

BD was the leading cause of uveitis in a multicenter registry in Turkey [25]. Subsequent studies carried out by various international institutions have consistently reaffirmed these findings and underscored the prevalent occurrence of uveitis as a manifestation of BD [26–29]. This ocular complication not only damages the patient’s overall well-being, but also poses a significant risk of permanent vision impairment.

Notably, there are complex connections that can be observed between BU and other BD symptoms. We can see a clear positive correlation between BU and both arthralgia and parenchymal neurological involvement. On the other hand, there is an opposite association when it comes to genital ulcers, gastrointestinal symptoms, and other systemic symptoms [30–33]. These complex interactions highlight the need for further investigation to better understand the multifaceted pathogenesis of BD.

Demographically, BU does not exhibit a specific age limitation, but it most frequently emerges in individuals between 25 and 44 years [34]. Pediatric presentations may deviate from the typical and display a more aggressive disease course [35]. The elderly may have milder symptoms, but they are susceptible to complications related to treatment due to existing comorbidities. Consequently, medications should be administered with caution in this population [36]. The interplay between BU and pregnancy remains enigmatic, necessitating cautious therapeutic considerations to protect fetal health [37].

Clinically, BU typically exhibits as a chronic, recurrent, bilateral non-granulomatous uveitis. The inflammation primarily affects either the anterior (11.1%) or posterior (28.8%) segment of the eye, although panuveitis, which involves inflammation in both segments concurrently, occurs more frequently (60.2%) [11, 26, 38, 39].

BD exhibits a significant predominance in males, with notable differences in prognosis based on gender. Specifically, male patients with BU experience a more rapid decline in visual prognosis, as indicated by a substantially 5-year and 10-year risk of losing useful visual acuity [8, 38]. These differences tied to gender may be attributed to the influence of testosterone on the regulation of neutrophils and T helper 1 cells (Th1), potentially shedding light on the increased morbidity observed in male BD patients [40].

### Clinical features and complications

BU is recurrent and presents as anterior uveitis, posterior uveitis or panuveitis (Table 3). It usually involves the whole eye, with bilateral involvement in 4 out of 5 patients. Men predominate and are at higher risk of losing useful vision than women. Although rare, isolated cases of anterior uveitis are predominantly reported among females [9, 38]. Smooth layered shifting hypopyon, diffuse vitritis, transient superficial retinal infiltrates, full-thickness retinal infiltrates, diffuse gliotic sheathing of retinal veins, peripheral occlusive periphlebitis, retinal hemorrhages, and fluorescein angiography revealing diffuse retinal capillary leakage, retinal capillary nonperfusion, and optic disc hyperfluorescence/leakage, are suggestive of BU [17, 41]. One of the concerning aspects of BU is its potential to cause irreversible vision loss, as well as damage to other organs, and even death. Younger males tend to have the poorest prognosis in these cases.

**Table 3:** Common clinical manifestations of BU

Clinical manifestation	Description
Anterior uveitis	Inflammation of the anterior segment of the eye, characterized by redness, pain, photophobia, and sterile hypopyon.
Posterior uveitis	Ocular inflammation, which encompasses the posterior segment of the eye, specifically the retina and choroid, can give rise to visual impairments and the presence of floaters.
Panuveitis	Ocular inflammation, which encompasses the posterior segment of the eye, specifically the superficial retinal infiltrates and precipitates particularly seen in BU patients.
Retinal vasculitis	Inflammation of the retinal blood vessels, leading to vascular occlusion and hemorrhage.
Optic disc edema	Swelling of the optic disc due to increased intracranial pressure or inflammation of the optic nerve.
Macular edema	Accumulation of fluid in the macula, leading to central vision loss and distortion.
Cystoid macular edema (CME)	A specific type of macular edema with cyst-like spaces in the macula, affecting vision.

### Auxiliary examinations

The diagnostic and therapeutic options for BU are enhanced by a wide range of retina imaging techniques that effectively outline its pathophysiological changes. Contemporary ophthalmological evaluations significantly rely on imaging methods such as color photography, B-scan ultrasonography, fundus fluorescein angiography, laser flare-cell photometry, and optical coherence tomography (OCT). These technologies introduce fast and accurate diagnostic possibilities, supported by the use of multiple imaging techniques [42, 43].

This suite of diagnostic tools ranges from classical color photography, enabling vitreous opacity and retinal infiltration documentation, to the technologically advanced OCT, illuminating macular afflictions and nuanced retinal layer alterations [44]. Furthermore, projection-resolved optical coherence tomography angiography (PR-OCTA) has illuminated the existence of macular circulatory anomalies in both eyes, irrespective of the BU is unilateral or bilateral [45]. Despite its ability to provide a detailed examination of retinal circulation, OCTA is limited in its ability to identify vascular leaks, which are typically detected through invasive methods that require the use of dyes [46].

The major component of BU is retinal inflammation, rather than choroidal inflammation. Indocyanine green angiography (ICGA) offers insights into choroidal inflammation and aids in distinguishing BU with predominant retinal affliction from conditions primarily impacting the choroid [47].

Fundus fluorescein angiography (FFA) provides valuable insights into both vascular and extravascular retinal that may not be apparent through fundus microscopy. These include vascular leakage, cystoid macular edema, and retinal vascular occlusion, among others [8]. FFA can detect fundus changes caused by posterior uveitis and panuveitis, determine the site and size of the lesion, and dynamically observe and evaluate the treatment effect. FFA is an indispensable tool in diagnosing and monitoring BU. FFA is particularly useful in identifying diffuse retinal capillary leakage, which presents as a fern-like pattern, indicating suboptimal response to therapy even during asymptomatic periods [42]. FFA is the gold standard for detecting and monitoring the leakage and occlusion of retinal vasculitis in BU patients [42, 48–50]. FFA findings also have prognostic significance. In active BU patients, disc neovascularization, macular window defect and macular ischemia indicate poor visual prognosis [49]. It not only provides important information on the vascular and optic disc leaks, but also provides crucial clues for clinical judgment. For example, FFA can distinguish the underlying cause (ischemia or pure inflammation) in the presence of neovascularization of optic disc or whether abnormal vessel clumps are shunt vessels or neovascularization in the scenario of retinal vascular occlusions. Although FFA has limitations, such as its invasive nature, potential allergic reactions, and a lack of quantitative measurements. It remains the golden standard among multimodal imaging in BU.

Laser flare-cell photometry (LFCM) has emerged as an effective and non-invasive tool for quantitatively evaluating intraocular inflammation in BU. It allows for precise detection and measurement of cells and proteins in the front part of the eye. Compared to traditional slit-lamp examinations, LFCM offers greater objectivity and accuracy, especially in identifying moderate to severe inflammation in the front part of the eye, which is a characteristic feature of BU [8]. Furthermore, the utilization of LFCM proves to be advantageous when it comes to the surveillance of continuous retinal vascular leakage in patients who are experiencing clinical remission. The reason for this correlation lies in the fact that the degree of flare observed through LFCM analysis is directly linked to the level of fluorescein angiographic leakage. Consequently, this connection reduces the necessity for frequent invasive procedures such as FFA [51].

Among them, fundus photography, FFA, and OCT continue to serve as the primary imaging modalities in BU.

Furthermore, thorough examination is being conducted on potential biomarkers such as *HLA-B51*、tumor necrosis factor-α (TNF-α), microRNAs, and specific sweat metabolites such as l-citrulline [52, 53]. In the case of untreated active BD, an increased risk of uveitis has been significantly associated with elevated serum IgA levels and antibodies against cardiolipin, β2-glycoprotein I, and prothrombin [54]. Although various autoantibodies and biomarkers associated with BU have been identified, their clinical significance remains to be further validated.

## Etiology and pathogenesis

### Genetic factors

The genetic composition of BD, situated within its epidemiological framework, deviates from the majority of systemic diseases. Interestingly, its worldwide presence intersects with the historical Silk Road. Present discussions on genetics firmly establish the significance of host genetic factors in determining susceptibility to BD (Table 4).

**Table 4:** Overview of genetic and environmental factors in BD

Factor		Description
Genetic factors	*HLA-B51*	Associated with BD and its uveitis manifestation
	*IL10, IL23R-IL12RB2* gene clusters	Genes involved in immune responses, linked to BD
	*ERAP1*	Involved in antigen presentation, associated with BD in individuals with the *HLA-B51* allele
	Other genetic loci	Implicated through genome-wide association studies
Environmental factors	Infections	Streptococci, Mycobacteria, Treponema pallidum, and others
	Oral and gut microbiome	Changes might play a role in disease onset and progression
	Smoking	May influence the onset and progression of the disease
	Geographic location	More prevalent in countries along the Silk Road

Although *HLA-B51* is not currently used as a diagnostic marker, it plays a significant role as a genetic contributor in BU. *HLA-B51* is positively correlated with ocular lesions but negatively correlated with gastrointestinal lesions [4, 55]. The precise reasoning behind the association of *HLA-B51* with BU is still a subject of scholarly discussion, particularly considering linkage disequilibrium. However, it is important to acknowledge that *HLA-B51* contributes to only a small portion (less than 20%) of the genetic risk, suggesting that there may be other genetic factors yet to be identified [56].

The endoplasmic reticulum aminopeptidase 1 (ERAP1) is pivotal in modulating peptide configurations within the endoplasmic reticulum (ER), thereby influencing the peptides presented by human leukocyte antigen (HLA) class I molecules. Certain *ERAP1* variants have shown a significant association with BD, especially in the context of HLA class I. A noteworthy revelation was the linkage of the *ERAP1* haplotype, Hap10, with BD. Remarkably, individuals carrying Hap10 and being homozygous for *HLA-B51* demonstrated an approximate elevenfold surge in disease susceptibility. Though Hap10’s strong linkage with BD is evident, its low prevalence ensures its limited influence on global risk. However, the combined impact of *HLA-B51* and Hap10 insinuates a profound genetic mechanism underpinning BD susceptibility. Recent studies utilizing genome-wide association studies (GWAS) and related functional annotations have brought attention to various susceptibility loci, such as *HLA-B51, HLA-A26, HLA-C0704* [5, 57], *CCR1* [58], *CCR1-CCR3, ERAP1, KLRC4, STAT4* [59, 60], *FUT2* [61], *IL12A* [62, 63], *IL10, IL23R-IL12RB2* [24, 64–66], *TRAF5, TRAF3IP2* [67], *ADO-EGR2, CEBPB-PTPN1, IL1A-IL1B, IRF8, LACC1, RIPK2* [68, 69], *PTPN2* [70], *STAT3* [71], *IL23R* [72, 73], *miR-146a* [74], and *miR-182* [75] (Table 5). However, the genetic aspects of BU are not yet fully understood. Despite GWAS identifying *HLA-B51* and other non-leukocyte antigen risk factors, a comprehensive genetic understanding of BU is still lacking, necessitating further investigation.

**Table 5:** HLA variants and susceptibility loci associated with BD

Locus	Chr	Variant	Allele/genotype			OR	95%CI	Ethnic group	Patients	Controls	*P* value	References
*HLA-B51*	6	-		-	5.586		5.151–6.657	Han Chinese	1015	4502	3.75E-190	[5]
*HLA-A26*	6	-		-	2.437		1.976–3.005	Han Chinese	1015	4502	9.77E-18	[5]
*HLA-C0704*	6	-		-	3.784		2.709–5.286	Han Chinese	1015	4502	6.07E-17	[5]
*LACC1*	13	rs9316059		T	0.687		0.607–0.777	Han Chinese	1238	1458	4.95E − 08	[69]
*CEBPB-* *PTPN1*	20	rs913678		C	1.32		1.22–1.42	Turkish, Iranian	2869	2605	1.43E − 12	[68]
*RIPK2*	8	rs10094579		A	1.302		1.149–1.474	Han Chinese	1238	1458	6.93E − 04	[69]
*ADO-* *EGR2*	10	rs224127		A	1.274		1.141–1.422	Han Chinese	1238	1458	3.77E − 04	[69]
*ERAP1*	5	rs17482078		TT	4.56		2.88–7.22	Turkish	2047	1908	4.73E − 11	[59]
*IL10*	1	rs1518111		A	1.45		1.34–1.58	Different geographic origins	2430	2660	3.54E − 18	[64]
*IL10*	1	rs1800871		TT	1.47		1.28–1.68	Han Chinese	1206	2475	5.88E − 08	[66]
*IL10*	1	rs3024490		TT	1.34		1.17–1.54	Han Chinese	1206	2475	2.80E-05	[66]
*IL23R-IL12RB2*	1	rs924080		TT	1.50		1.30–1.73	Han Chinese	1206	2475	2.03E − 08	[66]
*IL23R-IL12RB2*	1	rs12141431		CC	1.53		1.32–1.78	Han Chinese	1206	2475	2.18E-08	[66]
*IL23R-IL12RB2*	1	rs1495965		G	1.56		1.34–1.83	Japanese	612	740	1.2E − 08	[24]
*IL23R*	1	rs17375018		GG	1.86		1.39–2.49	Han Chinese	338	407	3.47E − 04	[73]
*IL23R*	1	rs17375018		G	1.57		1.25–1.98	Han Chinese	338	407	8.88E − 04	[73]
*IL23R*	1	rs11209032		A	1.48		1.21–1.82	Han Chinese	338	407	1.26E − 03	[73]
*IL23R*	1	rs11209032		AA	1.69		1.21–2.35	Han Chinese	338	407	0.024	[73]
*STAT4*	2	rs7574070		A	1.27		1.17–1.37	Turkish, Japanese	2659	2648	1.29E − 09	[59]
*STAT4*	2	rs897200		A	1.45		1.3–1.6	Han Chinese	703	2110	6.20E − 09	[60]
*TRAF5*	1	rs12569232		CG	0.617		0.481–0.790	Han Chinese	789	1601	1.08E − 03	[67]
*TRAF5*	1	rs12569232		GG	1.583		1.246–2.011	Han Chinese	789	1601	1.40E − 03	[67]
*TRAF5*	1	rs6540679		AG	1.750		1.467–2.086	Han Chinese	789	1601	3.60E − 09	[67]
*TRAF5*	1	rs6540679		GG	0.536		0.451–0.637	Han Chinese	789	1601	1.22E − 11	[67]
*TRAF5*	1	rs10863888		AG	1.343		1.127–1.601	Han Chinese	789	1601	0.009	[67]
*TRAF5*	1	rs10863888		GG	0.769		0.644–0.917	Han Chinese	789	1601	0.027	[67]
*IL1A-IL1B*	2	rs3783550		G	1.33		1.20–1.46	Turkish	1990	1779	2.12E − 08	[68]
*IRF8*	16	rs11117433		C	0.63		0.54–0.74	Turkish	1990	1779	2.73E − 08	[68]
*FUT2*	19	rs681343		T	1.36		1.19–1.56	Iranian	976	826	7.27E-06	[61]
*KLRC4*	12	rs2617170		C	0.78		0.72–0.85	Turkish, Japanese	2659	2648	1.34E − 09	[59]
*CCR1*	3	rs7616215		C	0.65		0.57–0.75	Iranian	973	828	6.01E − 09	[58]
*IL12A*	3	rs17810546		G	2.06		1.49–2.84	Different geographic origins	336	5843	9.31E-06	[62]
*IL12A*	3	rs1874886		A	1.61		1.36–1.89	Spanish	408	2122	1.62E − 08	[63]
*PTPN2*	18	rs7234029		AG	1.549		1.326–1.810	Han Chinese	906	2178	1.43E-06	[70]
*PTPN2*	18	rs7234029		GG	0.466		0.326–0.667	Han Chinese	906	2178	8.34E-04	[70]
*STAT3*	17	rs2293152		GG	1.712		1.238–2.369	Han Chinese	503	615	0.021	[71]
*miR-182*	7	rs76481776		C	0.60		0.49–0.73	Han Chinese	820	1800	1.81E-07	[75]
*miR-182*	7	rs76481776		CC	0.58		0.46–0.71	Han Chinese	820	1800	3.25E-07	[75]
*miR-146a*	5	rs2910164		CC	0.61		0.50–0.74	Han Chinese	809	1132	1.24E-05	[74]
*miR-146a*	5	rs2910164		C	0.75		0.66–0.85	Han Chinese	809	1132	1.33E-04	[74]
*CD40*	20	rs4810485		TT	1.98		1.38–2.83	Han Chinese	373	402	0.006	[269]
*CD40*	20	rs1883832		TT	1.73		1.22–2.46	Han Chinese	373	402	0.012	[269]
*CCR1/CCR3*	3	rs13084057		G	0.32		0.2–0.5	Han Chinese	653	1685	1.71E-07	[270]
*CCR1/CCR3*	3	rs13092160		C	0.28		0.2–0.4	Han Chinese	653	1685	6.50E-08	[270]
*CCR1/CCR3*	3	rs13075270		C	0.32		0.2–0.5	Han Chinese	653	1685	2.76E-07	[270]
*TGFBR3*	1	rs1805110		CC	0.617		0.441–0.863	Han Chinese	330	468	0.03	[271]
*SUMO4*	6	+ 438 C→T		C	1.7		1.3–2.2	Han Chinese	232	302	0.002	[272]
*TLR2*	4	rs2289318		CC	1.462		1.223–1.747	Han Chinese	838	1600	0.001	[273]
*TLR2*	4	rs2289318		C	1.470		1.260–1.714	Han Chinese	838	1600	6.89E-06	[273]
*TLR2*	4	rs3804099		CT	0.626		0.526–0.744	Han Chinese	838	1600	2.426E-06	[273]
*UBAC2*	13	rs3825427		T	1.5		1.2–1.7	Han Chinese	477	1334	6.9E-06	[274]
*UBAC2*	13	rs9517668		T	1.4		1.2–1.6	Han Chinese	477	1334	3.3E-04	[274]
*UBAC2*	13	rs9517701		G	1.4		1.2–1.7	Han Chinese	477	1334	2.9E-05	[274]

OR, odds ratio; CI, confidence interval

### Innate immune system

#### Neutrophils hyperactivity

**Fig. 1:**
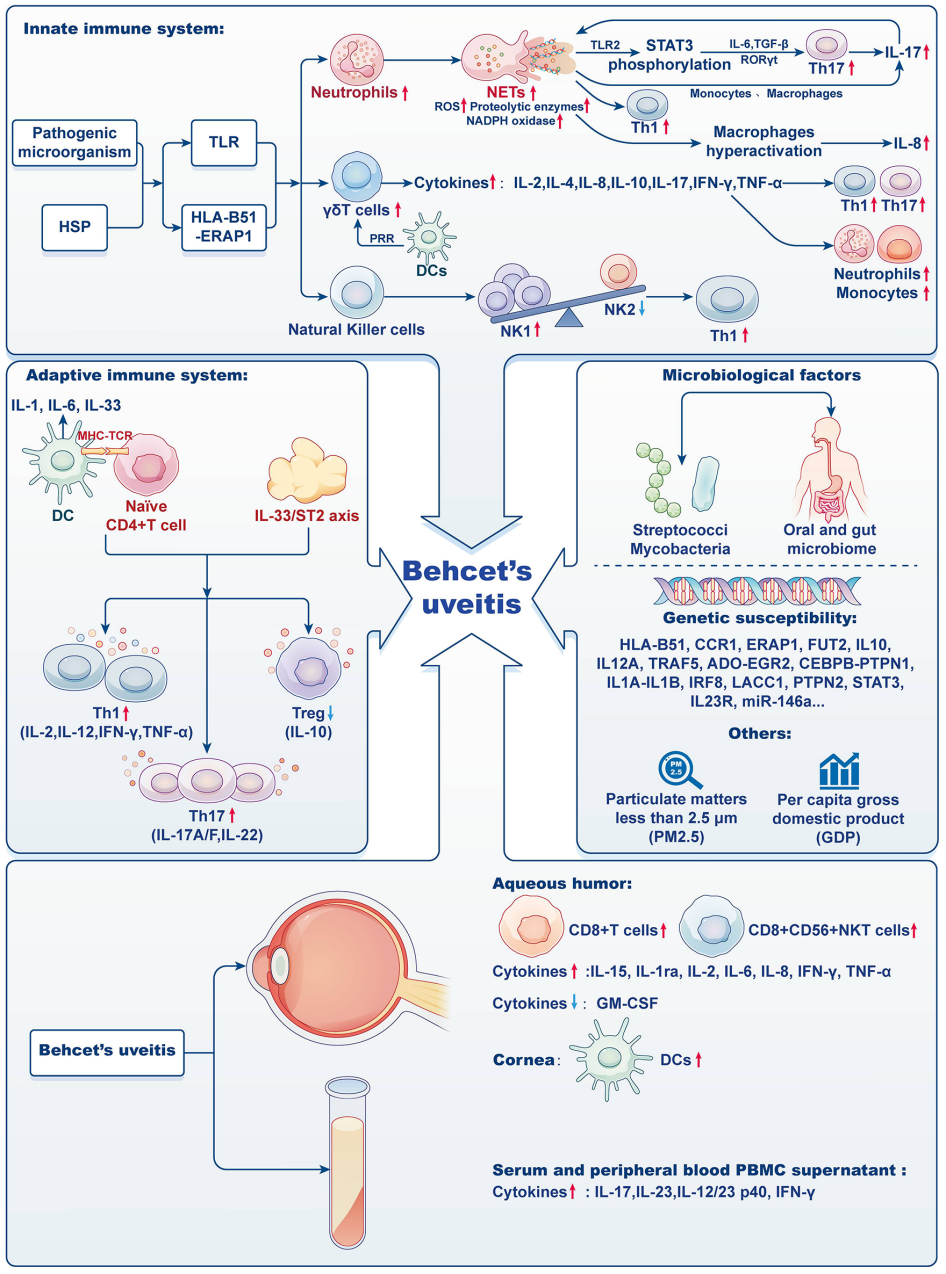
BU immunopathogenesis: Current understanding

The majority of leukocytes, known as neutrophils, serve as the foremost defense against infections. Although they perform a vital function in innate immunity, there is a potential for unintended harm to tissues, particularly in the presence of inflammation. This damage primarily occurs through phagocytosis, degranulation, and the release of neutrophil extracellular traps (NETs). Inflammation often leads to an increase in the number and lifespan of neutrophils [76, 77]. In the context of BD, there is a significant increase in neutrophil activity. This heightened activity may be associated with the *HLA-B51* gene and elevated levels of interleukin-17 (IL-17). The activated neutrophils have a propensity to aggregate in close proximity to blood vessels, whereby they release reactive oxygen species (ROS) and proteases. This process eventually culminates in the impairment of the vascular endothelium [2, 11, 78, 79]. The neutrophil-lymphocyte ratio (NLR) has been identified as a reliable biomarker to assess the extent of inflammation in BD and to evaluate disease severity [80, 81]. Further studies on BD patients have shown an enhancement in the oxidative burst and NADPH oxidase activities of neutrophils, resulting in increased production of ROS [82, 83]. Neutrophils in these patients also release various components such as NETs, DNA, extracellular reticulated DNA structures, histones, and myeloperoxidase (MPO) [84]. Notably, the histones from NETs play a dual role. They activate Th17 cells through intermediary cells like monocytes, and they directly induce STAT3 phosphorylation in T cells. This process results in the secretion of substances like IL-6, transforming growth factor-beta (TGF-β), and retinoid acid-related orphan receptor gamma t (RORγt). These substances further promote IL-17 production and the differentiation of Th17 cells, thereby amplifying NET formation (Fig. 1) [83, 85–87]. Additionally, elevated levels of NETs have been linked to increased differentiation of Th1 cells, specifically IFN-γ-producing CD4^+^ T cells. The subsequent increase in histone H4 and oxidized DNA within Th1 cells appears to trigger macrophage activation, resulting in enhanced production of IL-8 [88].

#### Hyperactivity of γδT cells

During the past 2 decades, there was a notable increase observed in the number of γδT cells in peripheral blood mononuclear cells (PBMCs) of BD patients. Normally, these γδT cells make up a small portion of the total T cells, ranging from 0.5 to 5%. It is worth mentioning that γδT cells possess characteristics of both innate and adaptive immunity, undergoing maturation through interactions with dendritic cells and pattern recognition receptors. They express molecules such as the inducible co-stimulator (ICOS) and CD40, and secrete various cytokines including IL-2, IL-4, IL-10, IL-17, IFN-γ, TNF-α, and granzyme (Fig. 1). These secreted molecules play a role in Th1/Th2 responses, link innate and adaptive immunity, and participate in autoimmune diseases.

Of particular interest, when it comes to oral pathogens, γδT cells may recognize these microbes through Heat Shock Proteins (HSP) and their T cell receptors. When neutrophils engulf these pathogens, γδT cells detect the resulting pathogenic compounds (such as (E)-4-hydroxy-3-methyl-but-2-enyl pyrophosphate) and release chemokines, notably CXCL8 (IL-8). The subsequent recruitment of neutrophils and monocytes, along with the induction of Th1 and Th17 responses, may contribute to the persistent inflammation observed in BD patients [89–92].

#### Elevated NK1/NK2 ratios

Natural killer (NK) cells, acting as cytotoxic lymphocytes in the innate immune system, exhibit their functionality independent of the constraints imposed by the major histocompatibility complex (MHC). Their primary function revolves around immunosurveillance while also exhibiting the ability to produce a diverse range of cytokines, including IFN-γ, IL-5, IL-13, GM-CSF, CCL3, and CCL4. In BD patients, abnormalities in the functionality of NK cells have been attributed to the presence of cytokines such as IL-10 and IL-15 [93–95].

What is particularly intriguing is that NK cells can be classified into two distinct types based on the cytokines they produce: NK1 and NK2 (Fig. 1). NK1 cells are primarily responsible to produce IFN-γ, whereas NK2 cells exhibit a broader role in immune modulation, producing cytokines such as IL-5 and IL-13. In active cases of BD, there is a prevalence of NK1 cells, whereas during periods of disease remission, NK2 cells dominate. This ratio between NK1 and NK2 cells provides valuable insights into the activity of the disease, as a higher NK1/NK2 ratio correlates with polarization towards a Th1 immune response and an increase in BD activity [96–98].

#### Dendritic cells: gatekeepers of Immune Response

Dendritic cells (DCs) are important antigen-presenting cells (APCs) that play a critical role in activating naïve T lymphocytes and contributing to both cellular and humoral immune responses. These cells play a crucial role in maintaining the balance of the immune system by connecting the innate and adaptive immunity. In normal physiological conditions, DCs can be found in various ocular tissues, including the central and limbal epithelia, basal lamina, and sub-basal nerve plexus layer [99–101]. However, the precise mechanism behind the inhibition of DC maturation in these conditions requires further investigation. DCs possess a distinctive capability in comparison to other APCs, as they can effectively initiate the activation of naïve T cells and facilitate their differentiation into Th1 and Th17 cells throughout the progression of a disease (Fig. 1). When exposed to chemokines or cytokines, immature DCs undergo maturation and migrate to lymph nodes, leading to increased expression of costimulatory molecules and MHC class II molecules [101–105]. The immature state of DCs is associated with the maintenance of immune tolerance. BU patients showed elevated expression of MHC class II and costimulatory molecules in the maturation profiles of peripheral blood DCs, even during periods of non-inflammatory activity. This finding suggests that the transition from an immature to a mature DC state may contribute to the chronic inflammation and relapse observed in BU. Moreover, BD patients exhibited a lower number of plasma cell-like DCs compared to healthy individuals, indicating that these cells may contribute to inflammation by migrating to target organs [106, 107]. During inflammation, DCs secrete IL-6, which influences the biological function of DCs and facilitates their activation [108]. The expression of programmed death ligand-1 (PDL1) and its transcription factor interferon regulatory factor I (IRF1) in DCs from active BU patients has been found to be decreased in recent studies. This decrease in expression is observed to correlate with the level of disease activity [109]. Confocal imaging studies consistently demonstrate an increased density of DCs in the corneas of BU patients, regardless of disease severity [110].

### Adaptive immune system

#### Role of T cells

T cells, central to adaptive immunity, have increasingly been a focus in the study of BU pathogenesis (Fig. 1). This increased interest can be traced back to the discovery in 2000 of clonally aggregated T cells in the anterior chamber of BD patients [111, 112]. The importance of T cell-mediated immune imbalances in BU has been underscored by recent transcriptome analyses conducted on iris samples. Specifically, the involvement of the T cell receptor signaling pathway and the prominence of helper T cell differentiation pathways highlight this connection. The lymphocyte-specific protein tyrosine kinase (LCK), which plays a critical role in T cell functions, has been identified as a key player in BU. The activated LCK signaling pathway and elevated active LCK expressions observed in BU indicate the potential of the *LCK* gene for therapeutic developments in BU treatment [113].

The increased expression of Th1/Th17-associated cytokines has led to the activation of the JAK/STAT signaling pathway, which has been observed in monocytes and CD4^+^ T cells [114–116]. The upregulation of gene expression leads to the activation of CD4^+^ T cells, resulting in their transformation into Th17 cells. This process is influenced by the release of inflammatory cytokines from monocytes. Subsequently, Th17 cells attract neutrophils and intensify the inflammatory reaction. Within the scope of BD, the signaling of serum amyloid-A is recognized as a pivotal element in guiding the differentiation of Th17 cells [117, 118]. RNA-seq studies of CD8^+^ T cells from BD patients have emphasized the importance of the cAMP-mediated signaling pathway in T cell activation. Interestingly, sustained elevation in cAMP levels tends to have an immunosuppressive effect [119, 120].

It has been shown that the affected regions are primarily infiltrated by CD8^+^ T cells. When comparing BU patients to those with other uveitis subtypes such as idiopathic recurrent acute anterior uveitis and Vogt-Koyanagi-Harada syndrome, it is observed that the aqueous humor of BU patients contains a higher concentration of CD8^+^ T cells, whereas CD4^+^ T cells dominate in the other subtypes. Conversely, skin samples from BD patients typically exhibit a higher presence of CD4^+^ T cells, along with fewer CD8^+^ T and CD56^+^ cells. This indicates a distinct intraocular immunomodulatory environment in BU that is characterized by a more aggressive inflammatory response. Furthermore, during active BU phases, CD8 bright CD56^+^ T cells secrete cytotoxic molecules such as dissolved protein perforin and surface FasL. These cells not only possess conventional CD8^+^ CTL cytolytic functions but also demonstrate NK-like cytotoxic activities. Another notable feature of BU is the significant increase in NKT cells in both the aqueous humor and peripheral blood. The primary subtype of NKT cells, CD8^+^ CD56^+^ cells, have the capability to exert strong cytotoxic effects, which can lead to the lysis of vascular endothelial cells through FasL- and perforin-dependent mechanisms, posing serious risks to vision. In contrast, patients with type 1 diabetes, a standard immune-mediated inflammatory disease, do not exhibit these characteristics. This emphasizes the uniqueness of CD8^+^ CD56^+^ cells as immune effectors, which may play a crucial role in the visual impairment observed in BU [121, 122]. The severe clinical manifestations of BU, in comparison to other types of uveitis, may be attributed to this factor.

The Th1/Th2 balance responses holds significant importance in the development of BU, with the Th1 response exerting a particularly strong influence. Th2 cells exhibit the capability to secrete cytokines with anti-inflammatory properties, namely IL-4, IL-5, IL-10, and IL-13. On the other hand, Th1 cells are distinguished by their ability to generate pro-inflammatory agents such as IL-2, IL-12, interferon, and tumor necrosis factor. It is noteworthy that BD patients typically exhibit elevated levels of Th1-related cytokines in their bloodstream [123, 124].

In the aqueous humor of BU patients, a notable increase in the concentrations of multiple cytokines is observed, which encompass IL-1ra, IL-2, IL-6, IL-8, IP-10, IFN-γ, and TNF-α. Conversely, the levels of GM-CSF are diminished. These cytokine levels correspond to the presence of inflammatory cells, particularly monocytes and neutrophils, emphasizing the potential role of the innate immune system in the development of BU. Interestingly, BU patients exhibit elevated concentrations of IL-15 in their aqueous humor, a characteristic not observed in individuals with other uveitis types such as human leukocyte antigen B27-associated uveitis, Vogt-Koyanagi-Harada syndrome, juvenile idiopathic arthritis, and idiopathic uveitis. IL-15, which possesses immunomodulatory properties, enhances the proliferation and activation of specific immune cells like NK cells, NKT cells, and CD8^+^ T cells. The prominence of CD8^+^CD56^+^ NKT cells in BU suggests their potentially detrimental role in the progression of the disease. Furthermore, active BU patients demonstrate a lack of the anti-inflammatory cytokine, IL-10, highlighting the unique immune characteristics of BU. The distinct presence of pro-inflammatory cytokines like IFN-γ and TNF-α in BU, compared to other forms of uveitis, suggests the potential for novel therapeutic strategies. By identifying the specific immune players and cytokines involved in the pathogenesis of BU, scientific advancements may lead to tailored treatments that alleviate symptoms and address the underlying cause of this immune disorder [125–128].

The significant presence of Th1 and Th17 cells in BD patients highlights the critical involvement of the adaptive immune system in both the onset and advancement of the disease. Transcriptomic studies have revealed an active NF-κB pathway in peripheral Th17 cells. Additionally, analysis techniques such as WGCNA and pathway enrichment have highlighted the activation of APCs in BD [11, 129]. The expression of IL-27, both at the mRNA and protein levels, is found to be reduced in the PBMCs and serum of active BD patients. IL-27 is known for its ability to suppress Th1 and Th17 cellular responses by inhibiting the expression of certain pro-inflammatory cytokines, including IL-1β, IL-6, and IL-23. Recent findings suggest that IL-27 can inhibit the differentiation of Th17 cells through the IRF-8 pathway [104], indicating that increasing IL-27 levels may help alleviate the inflammatory responses observed in BD patients.

Another crucial pathway involved in the development of immune-mediated disorders, including BU, is the IL-23/IL-17 axis [130, 131]. Increased levels of IL-23 prompt the transformation of naïve T cells into pathogenic Th17 cells. These cells then release pro-inflammatory cytokines like IL-17 A, IL-17 F, and IL-22, with the help of the intracellular JAK/STAT signaling cascade. Additionally, IL-23 contributes to the ongoing inflammatory response by upregulating its receptor, IL-23R [96, 132].

With regards to another cytokine, IL-33, a member of the IL-1 family, interacts with the ST2 receptor, resulting in the activation of MAP kinase and NF-κB. This interaction induces the production of pro-inflammatory cytokines, facilitates the differentiation of Th1 and Th17 cells, and is associated with the dysfunction of regulatory T cells (Tregs) [133, 134]. BD patients in the active phase display heightened levels of IL-33 and its soluble receptor ST2 (sST2). An intriguing observation is the correlation between ST2 levels and inflammatory markers such as erythrocyte sedimentation rate (ESR) and C-reactive protein (CRP) in BD patients. A potential reduction in serum ST2 levels has been observed following treatment with colchicine [135]. Furthermore, the investigation of single nucleotide polymorphisms within the IL-33 gene has revealed a correlation between the variants rs7044343 and rs2210463 and the occurrence of BU [136, 137].

#### Role of B cells

B cells are only a small part of the immune cells in BD, but the function of regulatory B cells (Bregs) is increasingly recognized. The primary function of Bregs is to produce anti-inflammatory cytokines, which are crucial for the proper functioning of regulatory T cells. By inhibiting T cell differentiation and suppressing autoimmune reactions, Bregs play a vital role in maintaining immune homeostasis. A noteworthy observation in BD patients is the substantial decrease in IL-10 mRNA levels within B cells. This finding opens up possibilities for developing novel therapeutic approaches for uveitis [138].

A notable characteristic of BD is the pronounced depletion of B cells, particularly Bregs. This depletion is primarily associated with a decline in CD27^+^ memory B cells expressing different immunoglobulin subsets, most notably IgM, IgG, and IgA, with a specific focus on CD27^+^ IgA^+^ B cells. It is speculated that these cells may migrate from the bloodstream to sites of inflammation. Considering their correlation with disease activity, these cells hold promise as potential biomarkers [139]. Furthermore, Breg counts have been found to be correlated with the severity of BD and ESR values. Interestingly, there appears to be a positive relationship between the number of Bregs and the dosage of corticosteroids administered to patients. However, recent studies suggest that the overall count of B cells and the number of Bregs remain consistent among BD patients, regardless of whether they exhibit symptoms of BU [140]. This indicates that further investigation is required to fully understand the precise role and impact of B cells in the pathogenesis of BU.

### Microbiological factors

Although there is no direct evidence linking BD to microbial infections such as viruses or bacteria, studies suggest that infectious pathogens may play a role in triggering the immune response associated with BD [2, 141]. Notably, studies have found an increased presence of Th17 cells in the peripheral blood of BD patients. It is hypothesized that alterations in bacterial composition and metabolism contribute to immune system disruptions, particularly in the balance between Th17 and Treg cells [142–145].

BD patients have shown a decrease in fecal concentrations of both Barnesiellaceae and Lachnospira, indicating a shift in gut microbial composition that may be connected to immune irregularities [146]. In an interesting study conducted by Shimizu et al. in 2018, fecal samples from 13 BD patients and 27 healthy individuals were analyzed. The findings revealed a significant increase in the relative abundance of Eggerthella lenta, along with six other bacterial species, in BD patients. The authors suggested that these gut microbes in BD patients could potentially induce immune anomalies by influencing nucleic acid and fatty acid synthesis, as indicated by the results of PICRUSt functional annotation analysis [147].

Stool samples from active BD patients have shown a decreased presence of bacteria that produce butyrate [148]. In another intriguing study, gut microbes from BD patients were transplanted into mice, resulting in weakened intestinal barrier strength and a reduction in three short-chain fatty acids (SCFAs) - butyric acid, propionic acid, and valeric acid. These SCFAs are known to stimulate Treg cells in the intestines and feces. Single-cell sequencing performed on these mice revealed evidence of activated neutrophils promoting the differentiation of Th1 and Th17 cells in specific lymph nodes and spleen cells [149, 150].

Other factors to consider in relation to BD include a history of tuberculosis (TB) infection and certain genetic predispositions associated with susceptibility to TB, which have been recognized as potential contributors to the onset of BD [151]. Additionally, elevated levels of antibodies against specific heat shock protein epitopes from Mycobacteria and Streptococci have been observed in BD patients. It is interesting to note that human heat shock proteins exhibit similarities to these epitopes, potentially leading to cross-reactive immune responses and subsequent autoimmune reactions [152, 153]. In another study, specific streptococcal strains were isolated from BD patients with extraocular myopathy [154].

### Others

A fascinating study conducted in mainland China investigated the relationship between air quality and the occurrence of uveitis. It was found that there was a strong association between exposure to particulate matters less than 2.5 μm (PM2.5) and the development of non-infectious uveitis and uveitis associated with systemic diseases, particularly in males aged 20 to 50. Interestingly, this association appeared to weaken over time, possibly due to increased biological adaptation or the implementation of individual protective measures [155].

Further investigation discovered that the positive association between increased PM2.5 levels and the occurrence of BU was exclusively observed in areas where the Per capita gross domestic product (GDP) exceeded the national average [156]. Japanese reports showing decreased incidence and severity of BD over decades, presumably associated with improved socioeconomic conditions [157, 158]. Also, a recent report from Turkey comparing BU patients with other noninfectious uveitis showed that BU patients were from GDP regions and had lower income [159]. Previous studies have emphasized the connections between economic development and the prevalence of immune and inflammatory diseases [160, 161]. Notably, economic growth itself showed an inverse relationship with the incidence of uveitis, particularly in male patients aged 20–50 years and markedly so in cases of BU [162]. The underlying reasons for this are not yet fully understood but could be associated with enhancements in mental and overall health stemming from a rise in GDP.

It raises contemplation that regions with a per capita GDP surpassing the national average might indeed experience a spike in uveitis incidence. A prevailing hypothesis attributes this to the concurrent rise in exposure to PM2.5 [156]. The integration of these findings has significant ramifications, providing insights that could aid the formulation of preventive measures and treatment strategies for uveitis in nations undergoing swift economic progression, especially those in the developing world.

Furthermore, the association between vitamin D and BU is gradually recognized. In individuals of the Chinese Han demographic, the *DHCR7* gene, which is associated with the vitamin D pathway, has emerged as a potential genetic predisposition for BD [163]. Recent research has emphasized the protective role of 1,25-dihydroxy vitamin D3 against BD. Interestingly, Vitamin D3 directly inhibits the differentiation of Th17 cells through the IRF-8 pathway [164]. A comprehensive study using Mendelian randomization, which included Chinese and Turkish samples with a total of 7,909 participants, demonstrated a correlation between elevated levels of 25-hydroxyvitamin D and an increased risk for BD. This suggests that caution should be exercised by clinicians when considering prolonged or high-dose vitamin D supplementation [6].

To summarize this section, an important characteristic of BD pathogenesis is the dysregulation of immune responses and the abnormal release of cytokines (Table 6). The available data provides substantial evidence to suggest that bacterial factors might have a substantial impact on the initiation of BD, thereby emphasizing the complex interaction between genetic predispositions and environmental factors in the advancement of the disease. These findings underscore the necessity for comprehensive risk assessment strategies in clinical settings to identify individuals with an elevated risk for BD. It is essential to further investigate this area to unravel the complex biological processes underlying these associations. This knowledge could potentially lead to tailored interventions for susceptible populations.

**Table 6:** Cytokines in BD

Cytokine	Species	Group	Sample Type	Regulation	References	
IL-1ra	Human	BU vs. HC	Aqueous	Up		[128]
IL-1β	Human	BD vs. HC	Saliva	Up		[275]
IL-2	Human	BU vs. HC	Aqueous Serum	Up		[124, 126, 128]
IL-4	Human	BD vs. HC	Serum	Up		[92]
IL-6	Human	BU vs. HC	Aqueous Serum	Up		[124, 128]
IL-8	Human	BU/BD vs. HC	Aqueous Serum Saliva	Up		[124, 128, 275]
IL-10	Human	BU vs. HC	Aqueous Serum	Down		[126]
IL-12	Human	BU/BD vs. HC	Serum Aqueous	Up		[124, 126]
IL-13	Human	BU/BD vs. HC	Aqueous NK cells	Up		[98, 128]
IL-15	Human	BU/BD vs. HC	Aqueous	Up		[126]
IL-17 A	Human	BU/BD vs. HC	Serum PBMC supernatants	Up		[124, 276–278]
IL-17 F	Human	BU/BD vs. HC	Serum PBMC supernatants	Up		[276–278]
IL-23	Human	BU/BD vs. HC	Serum PBMC supernatants	Up		[276, 278]
IL-27	Human	BU vs. HC	PBMCs PBMC supernatants Serum	Down		[104]
IL-33	Human	BD vs. HC	Serum	Up		[135]
IFN-γ	Human	BU/BD vs. HC	Serum PBMC supernatants Aqueous	Up		[92, 124, 126, 277, 278]
IP-10	Human	BU vs. HC	Aqueous	Up		[128]
TNF-α	Human	BU vs. HC	Serum Aqueous Saliva	Up		[124, 126–128, 275, 277]
TGF-β	Human	BD vs. HC	Serum	Up		[82, 86]
GM-CSF	Human	BU vs. HC	Aqueous	Down		[128]

HC, healthy controls; Up, up-regulation of cytokine levels; Down, down-regulation of cytokine levels

## Advances in therapies

Recent developments in therapeutic approaches for uveitis have highlighted the importance of a collaborative effort among ophthalmologists, rheumatologists, and internists, as emphasized by the 2018 European League Against Rheumatism (EULAR) guidelines. The primary goal is to effectively manage uveitis by reducing recurrent episodes and controlling inflammation. Timely intervention is crucial in cases of BU, as complications such as retinal ischemia and atrophy, optic atrophy, macular ischemia, macular edema, and further neovascular complications (vitreous hemorrhage, neovascular glaucoma) can lead to severe visual impairment or even blindness, and severely impacts the quality of life. Various factors, including medication adjustments, disruptions in circadian rhythm, fluctuations in emotions, and excessive consumption of tobacco and alcohol, have been associated with the recurrence of BU [165–169].

To avoid unnecessary complications, it is essential to closely monitor the outcomes of treatment and potential side effects during the management of BU. The choice of treatment strategies depends on the site of inflammation (e.g., anterior, posterior, or pan-uveitis), its severity, and underlying systemic conditions. The primary goals of therapy involve promptly suppressing inflammation, minimizing leakage of FFA, preserving vision, and preventing recurrence [170]. This section provides an overview of the latest advancements in pharmacological options for the treatment of BU (Table 7).

**Table 7:** Current and emerging therapies for BU: therapeutic actions and side effects

Therapy category	Treatment	Mechanism of action	Common side effects	Serious side effects
Supportive treatment	To prevent infection, it is advised to abstain from consuming stimulating foods, tobacco, and alcohol. Additionally, it is recommended to avoid any form of trauma, such as tooth extraction.	-	-	-
Corticosteroids	Topical and Systemic Corticosteroids	Anti-inflammatory and immunosuppressive via cytokine suppression and immune cell activity inhibition	Increased intraocular pressure, osteoporosis	Immunosuppression, delayed wound healing
Immunosuppressive agents	Azathioprine	Inhibits purine synthesis, reducing T-cell proliferation and cytokine production	Bone marrow suppression, gastrointestinal disturbances	Hepatotoxicity, increased infection risk
	Cyclosporine A	Inhibition of T-cell function by calcineurin	Increased uric acid, lipids, and blood pressure	Hepatotoxicity and parenchymal nerve involvement
	Methotrexate	Inhibits dihydrofolate reductase, decreasing DNA synthesis and immune cell proliferation	Hepatotoxicity, gastrointestinal upset	Bone marrow suppression, pneumonitis
	Chlorambucil	Alkylating agent that interferes with DNA replication	Nausea, vomiting, hair loss, bone marrow suppression	Risk of infection, increased risk of secondary malignancies
Biologics	-	-	-	-
TNF-alpha antagonists	Infliximab	Monoclonal antibody targeting TNF-α, a pro-inflammatory cytokine	Infusion reactions	Increased infection risk, development of antibodies
	Adalimumab	Monoclonal antibody against TNF-α	Injection site reactions	Increased infection risk, development of antibodies
	Golimumab	Monoclonal antibody against TNF-α	Injection site reactions, upper respiratory tract infections	Risk of serious infections, liver toxicity,
	Certolizumab pegol	Monoclonal antibody against TNF-α	Injection site reactions, upper respiratory tract infections	Risk of serious infections, liver toxicity,
IL-1 antagonists	Anakinra	IL-1 receptor antagonist reducing IL-1-mediated inflammation	Injection site reactions	Increased infection risk
	Canakinumab	Monoclonal antibody against IL-1β	Injection site reactions	Increased infection risk, development of antibodies
Janus Kinase (JAK) inhibitors	Tofacitinib	Inhibits JAKs involved in cytokine signaling	Headache, diarrhea	Blood disorders, increased infection risk
	Upadacitinib	JAK inhibitor	Upper respiratory tract infections, headache, nausea	Risk of serious infections, thrombosis, liver enzyme elevation
Emerging therapies	Secukinumab	Monoclonal antibody targeting IL-17 A	Injection site reactions	Upper respiratory tract infections
	Ustekinumab	Monoclonal antibody against IL-12 and IL-23	Injection site reactions	Upper respiratory tract infections

### Conventional therapies

#### Glucocorticoids (GCs)

have a pivotal role in the management of BU. In milder cases of BU with isolated uveitis and no systemic manifestations, oral GCs are suitable. For isolated anterior uveitis, topical GCs such as dexamethasone or betamethasone, along with ciliary muscle-relaxing agents, are beneficial. In cases where immediate inflammation reduction is needed at affected sites, pars plana or retrobulbar GC injections can be used. However, severe cases, particularly in younger males with early-onset disease, may experience anterior uveitis progressing to posterior forms, requiring systemic immunosuppression. These cases may need relatively high doses of systemic GCs to quickly control inflammation, followed by tapering to maintenance doses, ideally combined with immunosuppressants like azathioprine for posterior uveitis management. However, we found that inflammation was usually effectively controlled with relatively low doses of GCs in Chinese patients [1, 8, 171]. Intravenous high-dose methylprednisolone (IVPM) can improve visual clarity, reduce ocular inflammation, and prevent recurrences, often being more cost-effective than biologics [172]. In the severe cases, drugs like cyclosporine A or TNF-α inhibitors may be necessary, with interferon-alpha as an alternative for those who cannot tolerate TNF-α treatments. Topical steroid administration can be enhanced by the use of an intravitreal dexamethasone implant (Ozurdex), either alone or in combination with other treatments [173]. Another promising option for uveitis treatment is the intravitreal fludrocortisone implant (Iluvien, 0.19 mg). However, caution should be exercised when using topical steroids in individuals with glaucoma [174]. Local depot steroid injections should be avoided in patients with glaucoma or with a tendency to develop ocular hypertension with any steroid treatment.

Careful monitoring is essential, especially for growth effects in young patients, since long-term steroid use produces systemic side effects, including infections, hypertension, osteoporosis, and peptic ulcers. A collaborative approach involving ophthalmologists, rheumatologists, and internists is necessary for comprehensive patient evaluations, weighing the pros and cons of different therapies, and ensuring patient compliance.

### Immunosuppressants

Medications have emerged as reliable and cost-effective therapeutic options for the treatment of BU by suppressing the proliferation and function of immune cells. However, their tolerability and effectiveness have limitations, despite often being co-administered with steroids. Among these medications, antimetabolites such as azathioprine and methotrexate, as well as T-cell inhibitors like cyclosporine A, are currently used in BU for systemic immunosuppression with the goals of preserving vision and preventing recurrence [175, 176].

#### Azathioprine (AZA)

has been found to be effective in slowing down the progression of BU and reducing complications related to oral and genital ulcers and arthritis at a dose of 2.5 mg/kg/day [175]. A study involving 157 BU patients suffering from active posterior uveitis or panuveitis showed that a combination of corticosteroids (0.5 to 1 mg/kg/day) and AZA (2.5 mg/kg/day) led to total or partial remission in 93% of the patients, while also improving visual acuity. This allowed for a lower average dose of oral prednisone, which can lessen the risk of steroid side effects. In general, AZA has less severe side effects and is well tolerated in most patients, making it a reliable and efficient BU treatment. Its efficacy correlates with the severity of retinal vasculitis or vision loss and is enhanced with early administration [177]. It is also considered compatible for use in adolescent BU populations, often in combination with long-term steroid therapy [174].

#### Cyclosporine A (CsA)

is one of the most effective immunosuppressants for treating refractory BU and oral ulcers, skin lesions, and genital ulcers with long-term stable efficacy [171, 178, 179]. The daily dose is usually 3 to 5 mg/kg. Administration of 5 mg/kg/day CsA to active BU patients may significantly improve their vision within six months [8, 176, 180]. However, the application of CsA is restricted by its side effects, which include nephrotoxicity, elevated blood pressure, increased levels of liver enzymes, and gastrointestinal issues. Also, treatment of CsA increases the probability of parenchymal nerve involvement and elevates ALT/AST in BU patients, and the risk is greater when used alone than in combination with other drugs [181]. Other side effects of CsA are elevated uric acid, hyperlipidemia, and hypertension [179]. Therefore, careful dose adjustments tailored to individual patients are necessary.

#### Methotrexate (MTX)

is recognized as the least toxic immunosuppressive agent utilized in the management of posterior uveitis. A research study was conducted to assess the effects of a treatment regimen comprising prednisolone (0.5 mg/kg/day) and MTX (7.5 to 15 mg/week) on BU patients. Notable improvements were observed across posterior uveitis (PU), visual acuity (VA), and retinal vasculitis (RV), with PU displaying the most significant effectiveness. The total adjusted disease activity index (TADAI) diminished in 80% of the subjects [182].

#### Chlorambucil

, administered at a daily dose of 2 to 6 mg, has demonstrated potential in reducing BU relapses, controlling ocular inflammation, and improving systemic symptoms. Its benefits can persist even after discontinuation of the drug, and some patients may be able to reduce or stop steroid therapy. A retrospective study found that most BU patients responded to chlorambucil, and its use early in the disease resulted in better visual preservation. However, its dose-related side effects, like malignancy and myelosuppression, severely limit its application. Other side effects include nephrotoxicity, gastrointestinal reactions, leukopenia, infections, and temporary amenorrhea in women [183, 184]. One study reported that a short-term high-dose (mean duration: 23 weeks; mean total dose: 2.2 g) chlorambucil treatment was safer than a long-term application, with guaranteed efficacy. During the follow-up period, no malignancies were discovered [185].

In conclusion, while immunosuppressive agents offer a promising way to the management of BU, a cautious and individualized approach that balances efficacy and potential side effects is crucial. Collaboration among specialists is essential to tailor treatment plans to the specific needs of each patient.

### Biologics

Medications are monoclonal antibodies produced through genetic engineering that can rapidly improve disease and should be administered on the clinical characteristics of the patients [186].

#### TNF-alpha antagonists

The introduction of TNF-alpha (TNF-α) antagonists has brought about a significant change in the treatment of BU, leading to new therapeutic possibilities and a deeper understanding of the disease’s pathogenesis. Over the last two decades, these inhibitors have become the primary approach in managing severe uveitis manifestations in BD. Interestingly, BU patients tend to respond better to anti-TNF-α agents compared to those with idiopathic uveitis [187, 188].

In a groundbreaking development in 2017, adalimumab received approvals from both the US Food and Drug Administration (FDA) and the European Medicines Evaluation Agency (EMEA) for treating non-infectious uveitis. This approval was supported by numerous clinical studies consistently demonstrating the safety and effectiveness of TNF-α monoclonal antibodies in the long-term treatment of BU [189–192]. Furthermore, adalimumab and infliximab have comparable efficacy in the treatment of refractory BU. This remains true whether they are used as standalone treatments or in combination with other therapeutic agents like azathioprine (AZA) and methotrexate (MTX). These findings highlight the exceptional potential of anti-TNF-α agents in the treatment of BU [192, 193].

##### Adalimumab (ADA)

, is a recombinant IgG1 monoclonal antibody that is specially designed to target TNF-α. It is a completely humanized antibody that exhibits a strong binding affinity for p55 and p75 TNF receptors. Through its binding to these receptors, ADA effectively suppresses the activity of both the membrane-bound and soluble forms of TNF-α [194]. This inhibitory action is crucial in the treatment of non-infectious intermediate, posterior, or panuveitis, especially in cases where conventional therapeutic strategies have proven to be ineffective.

To initiate treatment, a subcutaneous dose of 80 mg is usually administered, followed by a maintenance dose of 40 mg every other week. Clinical outcomes have shown positive results with ADA therapy. Notable improvements include a significant reduction in intraocular inflammation, an enhancement in best-corrected visual acuity (BCVA), and a decrease in macular thickness as measured by OCT. Additionally, recurrence rates have been observed to decrease, indicating the safety and efficacy of ADA in the management of BU. The efficacy of ADA is not limited to patients who are newly introduced to the drug. Even patients who have failed primary anti-TNF-α treatments have experienced benefits when switched to ADA or other alternative anti-TNF-α agents [193, 195]. It is important to note that both ADA and infliximab can be used for the long-term treatment of BU, as their efficacy does not change even when used concurrently with DMARDs (Disease-Modifying Antirheumatic Drugs). ADA has also demonstrated efficacy in severe refractory BU patients, even in the presence of adverse prognostic indicators [196, 197]. While ADA is generally well-tolerated, some patients may experience localized reactions at the injection site [198]. Recent research highlights the potential of combining ADA with conventional therapies, particularly in the treatment of refractory BU-induced retinal vasculitis (RV). These combination treatments have shown superior outcomes compared to traditional therapies alone. Although patients on ADA often achieve stable long-term results, there may be a slightly increased risk of adverse events. Therefore, an individualized and flexible approach is recommended when administering ADA to ensure optimal outcomes [199, 200].

##### Infliximab (IFX)

is a chimeric monoclonal antibody made up of both human and mouse components. It is engineered to have a high binding affinity for both soluble and membrane-bound forms of TNF-α. In 2001, Sfikakis et al. pioneered the use of a single infusion of IFX (5 mg/kg) to treat five patients with recurrent BU, all experiencing rapid and sustained remission with no notable side effects during the observation period [201]. Subsequent clinical trials have consistently emphasized the potential effectiveness of IFX in the management of patients with refractory BU. It has been identified as a primary treatment for refractory retinitis caused by BD. The administration of IFX requires careful optimization, especially for patients who have achieved remission. For those who experience a relapse, the recommended treatment regimen involves the continuation of intravenous IFX at a dose of 5 mg/kg every eight weeks [202]. Infusion intervals are shortened in patients who experience relapse during IFX treatment and higher doses (more than 5 mg/kg) can be administered as well. A notable feature of IFX is its rapid therapeutic effect. Just a single infusion at a dose of 5 mg/kg has been observed to almost resolve all ocular manifestations of BU entirely within 28 days. This encompasses the resolution of retinal vasculitis, the disappearance of persistent symptoms such as macular cystoid edema, and significant improvements in visual acuity. The overall recurrence rate also drops substantially. While some patients do experience relapses, administering IFX again post-relapse has shown to be effective [203, 204]. The efficacy of IFX as a monotherapy might be slightly inferior compared to when it’s combined with CsA. Interestingly, after discontinuation of IFX, approximately 40% of patients maintained remission of their ocular inflammation for up to three years. This suggests that IFX offers a prolonged therapeutic effect, and discontinuation might be feasible for patients who demonstrate stable inflammatory control over an extended period [205]. A long-term (decade-long) clinical study further attested to the efficacy of IFX in managing BU. Patients showed significant visual function improvements and had a reduced incidence of ocular complications, such as glaucoma, during their follow-up. IFX also exhibited potential in managing overall BD symptoms, beyond just the ocular manifestations [206]. For patients where Interferon-alpha (IFN-α) therapy proves ineffective, IFX emerges as a viable alternative [207]. An important observation was that patients with uveitis symptoms for less than 18 months derived more benefits from IFX treatment. This underscores the potential advantages of initiating IFX therapy early in the disease course [208, 209]. However, caution is warranted when considering discontinuation of IFX. Even in patients who achieved long-term remission, extraocular manifestations, such as recurrent oral ulcers, were prevalent a year post-IFX discontinuation [210]. While IFX is generally well-tolerated, mild infusion reactions are common adverse events. However, clinicians should be wary of the potential for more serious complications, including severe infections (like reactivation of latent TB) and malignancies [198, 202].

A comparative analysis between IFX and ADA in the treatment of refractory BU has demonstrated the effectiveness of both drugs. Both IFX and ADA have shown positive therapeutic effects, but a multicenter study with one-year follow-up showed that ADA had better results in terms of improvement in anterior chamber inflammation, improvement in vitritis, and BCVA [198].

**Golimumab (GOL)**, a recent addition to the anti-TNF armamentarium, stands out due to its lower likelihood of inducing neutralizing antibodies compared to IFX and ADA [211]. Five refractory BU cases (8 eyes) treated with standard doses of GOL (50 mg every four weeks) were followed up for 12 months, and 7/8 (87.5%) eyes were found to have complete control of intraocular inflammation, demonstrating that GOL treatment significantly reduces the number of BU recurrences and rapidly regresses active retinal vasculitis (RV) [212]. GOL also demonstrated significant reductions in macular center thickness, vitreous opacity grading, and anterior chamber cell grading. The results of these studies suggest that GOL has the potential to become a mainstay for the treatment of refractory BU, especially in patients who have not received prior TNF therapy. Mild adverse effects included elevated liver enzymes, fatigue, and a rash [213].

##### Certolizumab pegol (CZP)

has a therapeutic effect on refractory BU that is outside the drug indications. There was a significant reduction in relapses after initiating GOL or CZP, with no discernible difference in the two drugs’ efficacy or survival. When other anti-TNF-α drug treatments are ineffective, GOL and CZP are alternative treatment options that can significantly reduce the frequency of relapses and preserve visual function [214]. CZP distinguishes itself from other anti-TNF-α drugs by lacking an Fc region, which interacts with the neonatal Fc receptor (FnRn). This structural difference results in a lower rate of placental transfer, making CZP a safer therapeutic option during pregnancy [215]. Studies have shown that CZP effectively reduces intraocular inflammation and preserves vision during gestation without causing harm to the newborn [216].

There is growing interest in the localized management of uveitis through intravitreal injections of anti-TNF agents. Early investigations suggest that this mode of administration allows for the rapid attainment of therapeutic drug levels in the eye. Furthermore, intravitreal injections of both IFX and ADA have demonstrated a favorable safety profile, exhibiting neither toxicity nor immunogenicity. Despite these advantages, the short duration of action of intravitreal IFX necessitates repeated injections. As BD is a systemic condition, a comprehensive assessment of the efficacy and safety of intraocular versus systemic medications is needed. It is possible that future treatments may incorporate both local and systemic anti-TNF medications [194].

BU patients who have achieved remission with repeated anti-TNF therapy may have their dose gradually reduced or their injection interval extended. TNF-α inhibitors can reduce the oral dose of GC, a phenomenon known as the steroid-sparing effect [193]. The steroid-sparing effect is an additional benefit of anti-TNF therapy. Patients who achieve remission with repeated anti-TNF doses may be able to reduce the dosage or extend the dosing interval. By effectively reducing inflammation, TNF-α inhibitors can decrease or eliminate the need for corticosteroids, thereby reducing the risk of steroid-related side effects. This potential to decrease BU recurrence compared to traditional treatments offers hope for preventing irreversible vision loss [217]. However, anti-TNF therapy is not without its risks. Systemic inhibition of TNF can lead to severe infections, including the activation of TB or reactivation of hepatitis B virus [218]. There are other potential side effects associated with the use of TNF-α, including exacerbation of heart failure, neuro-demyelinating lesions and dyslipidemia [219, 220]. Therefore, it is crucial for clinicians to use TNF drugs judiciously and regularly monitor their patients. The mechanism by which TNF-α functions likely involves the activation of macrophages, interactions with T cells, and T-cell-driven B cell responses.

#### Interferon-alpha

Interferon serves as a powerful immunomodulatory agent that has significantly changed the therapeutic landscape for BU [221, 222]. Its efficacy may be related to the decrease in dysfunctional Treg cells, Th17 cells, CD4^+^ T lymphocytes, and increase in IL-10 [223–225]. In recent guidelines, the EULAR advocates using high-dose corticosteroids, infliximab, or IFN-α for BU patients presenting with severe ocular manifestations. Based on treatment response following conventional therapy, Eser-Ozturk et al. split 25 BU patients receiving IFN-α into three groups: non-responsive group, complete remission group, and partial remission group. IFN-α was delivered at a dose of 6 million units (MU) daily over one week, then 3 MU per day. After clinical remission, IFN-α 3 MU was administered as a maintenance dose every other day. Assessing BCVA, central macular thickness (CMT), and FFA, 21 patients in total, accounting for 84% of the study population, exhibited enhancements after IFN-α therapy administration. Satisfactory results were obtained within a month, with rapid resolution of active inflammation and improvement of mean BCVA and CMT in all patients. Inflammatory episodes were never observed in the group with full remission, whereas increasing the IFN-α dose was effective in the partial remission group [226]. Besides being effective in treating ocular manifestations, IFN-α has proven to be particularly effective for BU patients with concomitant macular edema [227]. One of the noteworthy attributes of IFN-α therapy is the potential to achieve long-term remission post-drug withdrawal, indicating its potential for sustained therapeutic effects. Initiating IFN-α therapy early in the disease trajectory appears to yield better outcomes [226].

A recent study has shown that the co-inhibitory molecule PDL1 is upregulated by IFNα-2a in dendritic cells of BU patients in an IRF1-dependent manner and that PDL1 mRNA expression levels are linked to the treatment efficacy. Treatment with IFNα-2a led to CD4^+^ T cell apoptosis, without any significant changes in Treg frequency, and resulted in decreased Th1 and Th17 frequency and reduced levels of IFN-γ and IL-17. Suppression of the Th1/Th17 immune response corresponded to uveitis remission. Moreover, IFNα stimulated IL-10 secretion by CD4^+^ T cells in BU patients, which then hindered IL-17 secretion by PBMCs. In conclusion, the therapeutic benefits of IFNα-2a in BU are mediated by dendritic cells and CD4^+^ T cells [109].

In a monocentric retrospective investigation, Shi et al. incorporated 30 patients afflicted by refractory BU, who underwent IFN-α2a therapy at Peking Union Medical College Hospital between February 2015 and June 2018. Utilized as an adjuvant to traditional treatment in patients with poor prognosis led to treatment success in 26 individuals, representing 86.7% of the cohort. Throughout the follow-up period, most patients could achieve a reduction in steroid hormone and immunosuppressant dosage, or even complete discontinuation of immunosuppressant use, with a significant decrease in ocular inflammatory recurrence. No unresolved adverse drug reactions were observed [228].

Another study involving 36 patients with severe BU manifestations showed the efficacy of IFN-α in alleviating vasculitis, papillitis, and macular edema. There was also a notable decline in the mean annual recurrence rate per patient, even post-discontinuation of the interferon therapy [229, 230]. Pegylated interferon is a derivative of IFN-α that improves the solubility of IFN protein and prolongs its half-life. Therefore, in cases where IFN-α needs to be used three times a week, peg-IFN-α only needs to be used once a week at a frequency sufficient to ensure the therapeutic effect. A small case series involving four patients with severe refractory BU found that peg-IFN-α has a potential long-term effect for the treatment of severe uveitis, reducing the number of injections, improving the quality of life of patients, and improving treatment adherence [231]. There were notable variations in the management of ocular inflammation and good patient tolerability comparing the average number of episodes, visual acuity, ocular inflammation, FA score, disease activity, and side effects between IFX and IFN-α for treating refractory BU. When comparing IFX and IFN-α for the treatment of refractory BU, it was observed that IFN-α is a favorable therapeutic choice for BU patients who do not respond to conventional therapies, even considering its elevated risk of side effects [207, 227].

A direct comparison between IFNα-2a and corticosteroids versus CsA and corticosteroids over a 12-month period showed superior outcomes with IFN-α treatment, with significantly lower BOS24 scores, greater rates of BCVA, full remission, and more stable remission of intraocular inflammation. The advantages of IFN-α surpass those of CsA, which had a short-lived effect, a greater incidence of side effects, and a notable absence of significant steroid-sparing effect [179].

While IFN-α is typically well-tolerated, certain patients may encounter side effects like fever, fatigue, muscle pain, headache, and other flu-like symptoms. Other rare side effects include mild bone marrow suppression and elevated liver enzymes. However, with a multidisciplinary approach, most side effects are reversible [226].

Collectively, IFN-α has emerged as a reliable and effective treatment option for refractory BU. Its ability to reduce the need for steroids and other immunosuppressants, combined with its superior outcomes when used in the initial stages of the disease, solidifies its position in the therapeutic arsenal against BU.

#### CD20 antagonists

##### Rituximab

has been explored as a therapeutic option for B cell-mediated diseases by targeting the CD20 antigen found on B cell surfaces. In the case of refractory BU, rituximab has shown promising treatment outcomes. Specifically, two doses of 1000 mg each of rituximab, administered 15 days apart, have significantly reduced uveitis activity and associated symptoms in patients with retinal vasculitis and edema [232]. However, the current data on the utility of rituximab for BU is still insufficient to definitively address the side effects associated with uveitis treatment [233].

#### CD52 antagonists

**Alemtuzumab** has also been investigated for its potential in BU treatment. Alemtuzumab is directed against CD52, a protein found on the surface of lymphocytes and macrophages. This targeting results in the depletion of T cells and, ultimately, the reconstitution of immune function within the CD4^+^ cell subset [234]. Studies have shown that alemtuzumab can induce remission, reduce steroid dependency, and generally be well-tolerated in BU patients. However, careful assessment is required before its administration due to potential side effects like lymphopenia and thyroid function abnormalities [235]. Additionally, alemtuzumab has demonstrated efficacy in treating non-infectious uveitis associated with other conditions like multiple sclerosis [236].

#### IL-1 antagonists

##### Anakinra (ANA) and Canakinumab (CAN)

, as IL-1 antagonists, may serve as a treatment option for BU patients exhibiting resistance to traditional therapy and/or presenting contraindications for TNF-α inhibitors, such as latent TB or chronic/active infectious disease. A retrospective examination of 36 BD patients treated with ANA or CAN, conducted by Fabiani et al., revealed that IL-1 blockade demonstrated favorable therapeutic efficacy in BU and BD patients with extended disease duration. The therapeutic impact of ANA (100 mg/day) or CAN (150 mg/8 weeks) proved to be both rapid and enduring [237]. The results of observational studies have shown that IL-1 inhibitors can treat refractory BU and have excellent safety [238]. ANA is an interleukin-1 receptor antagonist, whereas CAN operates as an anti-interleukin-1 beta antibody. Fabiani et al. explored the roles of ANA and CAN in 19 patients with refractory BU (involving 31 affected eyes). They found that IL-1 inhibition therapy contributed to a considerable reduction in recurrence rates 12 months post-treatment initiation compared to the same duration before treatment initiation. Additionally, it significantly ameliorated retinal vasculitis in both short and long-term contexts, as well as reduced the average steroid dosage. However, the combination of IL-1 inhibitors and immunosuppressants did not enhance efficacy. Patients receiving concomitant DMARDs exhibited a higher rate of BU relapse relative to those undergoing monotherapy. In conclusion, ANA and CAN are effective and safe treatment options for BU, significantly reducing ocular inflammatory response activity, alleviating retinal vasculitis, preventing visual impairment, and significantly reducing steroid dose [239].

##### Gevokizumab

, a recombinant humanized variant monoclonal antibody, impedes IL-1 receptor activation by binding to human interleukin (IL)-1β. A phase II investigation involving the administration of 30 or 60 mg of gevokizumab intravenously or subcutaneously every four weeks to BU patients experiencing recent acute ocular deterioration or at risk thereof, yielded rapid control of intraocular inflammation within one week, accompanied by favorable steroid-sparing effects [240]. Despite demonstrating a good safety profile in the expanded study, gevokizumab failed to substantially reduce the risk of visual deterioration, leading to the study’s primary endpoint not being met. Consequently, it is not advised as a BU treatment based on the current, somewhat promising results [241, 242]. As such, further exploration of IL-1β pathway regulation in BU patients is warranted.

Furthermore, the administration of IL-1 inhibitors has the advantage of reducing the dosage of GC, leading to steroid-sparing effect. This reduction is beneficial as it minimizes the systemic and ocular-related side effects associated with prolonged GC use.

#### IL-6 antagonists

##### Tocilizumab (TCZ)

, a fully-humanized monoclonal antibody, acts on both membrane-bound and soluble IL-6 receptors, presenting a promising approach for treating BU, especially in instances where the condition is refractory. Inhibiting IL-6 can suppress the production of autoantibodies and rectify imbalances between autoantigen-specific Th17 and/or Th1-Treg [243]. TCZ has produced rapid and long-term improvements in ocular manifestations of BU, including anterior chamber cells, vitreous inflammation, chorioretinitis, and retinitis, but has limited efficacy in treating extraocular manifestations. Tocilizumab application may reduce the dose of GC and produce a steroid-sparing effect [244]. Macular cystoid edema, the most common complication of BU, can resolve rapidly after the first TCZ infusion, indicating that TCZ has great therapeutic potential for patients with refractory uveitis macular edema. Mild and rare treatment-related side effects include fatigue, chest tightness, transient elevation of serum LDL cholesterol (low-density lipoprotein) levels, and leukopenia [245]. In a recent multicenter retrospective observational research, tocilizumab showed higher efficacy against BU than IFX and ADA at six months of treatment and induced complete remission of macular edema in uveitis patients [246]. It demonstrates that TCZ is a secure and successful therapy for BU. TCZ also has a therapeutic effect on arthritis and phlebitis in BD but is ineffective in treating oral/genital ulcers and skin mucosal manifestations [247].

#### IL-17 a antagonists

##### Secukinumab

, a human monoclonal antibody with a high affinity for interleukin-17 A, has been determined to be ineffective in the treatment of BU. In a phase III randomized controlled trial involving 118 BU patients, administering subcutaneous injections of secukinumab–initiated with 300 mg dose every two weeks, followed by a maintenance dose of 300 mg every four weeks—did not succeed in reducing the recurrence rate of uveitis or improving BCVA. The primary treatment endpoint was not fulfilled, and it was also shown that the treatment group experienced more non-ocular adverse events than the control group did [248]. A proof-of-concept study found that compared with 300 mg subcutaneous injection 4 times every two weeks, secukinumab 30 mg/kg twice intravenously every four weeks may be necessary to deliver secukinumab in therapeutic concentrations. High-dose intravenous secukinumab has shown positive efficacy in patients with active chronic noninfectious uveitis who required corticosteroid-sparing immunosuppressive therapy [249]. However, there are also cases of new-onset BD reported in ankylosing spondylitis patients treated with secukinumab [250]. Secukinumab is currently not used to treat uveitis in BD.

#### IL-23 antagonists

##### Ustekinumab

is a fully-humanized monoclonal antibody designed to target the shared p40 subunit of IL-23 and IL-12. It has been observed that patients with active BU present higher serum levels of IL-23 compared to those with the inactive form of the disease [132]. Although there are limited studies and reports available on the efficacy of ustekinumab for treating BU, there exists a case report that details a successful instance of treating a BU patient. In this case, the patient received subcutaneous injections of ustekinumab, administered at 45 mg at weeks 0 and 4, and subsequently every 12 weeks, demonstrating effectiveness over a 3-month duration [251]. Typical side effects encompass nasopharyngitis, headache, abdominal pain, and joint pain [252].

#### Janus kinase inhibitors (JAKi)

##### Tofacitinib

functions as a JAK1/3 inhibitor, influencing both innate and adaptive components of the immune system. This mechanism is achieved by blocking the signaling pathways of multiple cytokines and interferons, such as IL-2, IL-4, IL-6, IL-23, IFN-γ, and IFN-α, leading to the regulation of immune responses. As a small molecule, tofacitinib possesses the potential to traverse the blood-retinal barrier with greater efficacy compared to conventional drugs. Tofacitinib 5 mg given twice daily significantly improves BCVA by reducing retinal leakage and decreasing recurrence, is well tolerated, and is therefore expected to be the first choice for treating BU in the future [253].

##### Upadacitinib

, a selective inhibitor targeting JAK-1, has garnered attention for its therapeutic potential in BU. In a recent study, the efficacy of upadacitinib was investigated in BU patients who exhibited inadequate responses to conventional therapies and anti-TNF-α treatments. Following the administration of upadacitinib to one adult and one pediatric patient, notable improvements were observed. Both patients experienced enhancements in visual acuity, effective control of intraocular inflammation, and resolution of macular edema. Importantly, no severe adverse events were reported during the follow-up period, underscoring the promising safety profile of upadacitinib in the management of BU [254].

JAKi offers a new option for BU patients, particularly those whose uveitis has not responded well to conventional and biological DMARDs [255].

### Chinese medicines

BD belongs to the “fox confusion disease” category in Chinese medicine. According to Chinese medicine, the formation of BD is internally related to the deficiency of spleen qi caused by factors such as physical constitution, diet, and emotion. Externally, the disease develops due to the invasion of the body by the evil of dampness and heat.

Longdan Xiegan Decoction (Lobelia, Gardenia, Scutellaria, Mouton, Zedoary, Plantago, Bupleurum, Glycyrrhiza, Angelica, Radix et Rhizoma) can regulate CD4/CD8 and Th17/Treg balance, thus effectively alleviating inflammation in experimental autoimmune uveitis (EAU) eyes and regulating systemic immune status [256].

Berberine, an isoquinoline alkaloid with a unique tetracyclic structure isolated from Chinese herbal medicine, has been found to have immunomodulatory effects in several inflammatory models [257, 258]. In uveitis, it has been confirmed that it can significantly ameliorate the BU and EAU, and there are two possible pathways. One is to affect genes belonging to chromatin remodeling and immune-related pathways, directly acting on T cells or indirectly through DC to regulate Treg/Th17 balance. The second is to increase the number of immunomodulatory bacteria in the gut microbiome [259–261].

These studies open new avenues of thought. Chinese medicine or natural products possess unique inherent principles for treating the disease and other T cell-related conditions. It is anticipated that a unified standard for Chinese medicine treatment of BU will emerge, potentially offering unforeseen therapeutic benefits in managing BU.

### Others

Progranulin (PGRN), an immunomodulatory molecule, has been observed to be downregulated during active disease phases in BD patients. Preliminary studies in animal models suggest that PGRN has the potential to alleviate EAU by reducing Th1 and Th17 cell populations, while simultaneously promoting the polarization of Treg cells. These findings suggest that PGRN may become a potential therapeutic target for BU in future investigations [262].

In addition to pharmaceutical treatments, dietary modifications are emerging as potential therapeutic strategies for BD. More specifically, diets rich in butyrate have shown promising effects by reducing the production of ROS in lymphocytes, monocytes, and neutrophils among BD patients. Furthermore, these dietary adjustments have been associated with decreased levels of CRP and increased overall plasma antioxidant capacity. These modifications contribute to a balanced inflammatory response, decreased disease activity, and reduced reliance on steroids [263]. The “hygiene hypothesis” holds that BD patients are more likely to live in poor sanitary conditions, characterized by lower monthly income, a history of parasites, use of dried cow dung as fuel, less bathing or brushing, and close contact with pigs and pork [264, 265]. The oral health status of BD patients is often worrisome, such as oral infection, need for tooth extraction, caries, loss of teeth, and an elevated plaque index score, which may be potential mediators of disease severity [266–268]. Hence, improving oral and personal hygiene may be beneficial.

These findings emphasize the importance of a comprehensive approach to managing BD, which should integrate dietary, lifestyle, and pharmacological interventions to optimize patient outcomes.

## Conclusions and future work

Taken together, uveitis is one of the primary clinical signs of BD. This manifestation is attributed to a combination of immune dysregulation, genetic predispositions, and the involvement of microbial elements. These elements have the potential to trigger aberrant immune responses that ultimately lead to the onset of the disease.

The treatment of BU extends beyond conventional pharmacological interventions. Lifestyle choices, environmental factors, and dietary habits have been acknowledged for their ability to influence disease activity, presenting a multi-faceted approach to managing the condition. Various molecular and cellular targets, such as the *LCK* gene, *ERAP1*, the balance of Th17 and Treg cells, the JAK signaling pathway, PGRN, and key cytokines like IL-17 and RORγt, have been identified as potential avenues for innovative therapeutic interventions.

Biologics have revolutionized the management of refractory BU due to their targeted mechanisms of action. However, there is still ongoing work to optimize BU treatment. Some current medications, while effective, have limitations, ranging from incomplete disease control to the occurrence of unwanted side effects. This emphasizes the urgent need for therapeutic options that not only offer improved efficacy but also greater tolerability for patients.

The future of BU treatment looks promising. With continued research and a deeper understanding of the disease’s pathophysiology, the medical community is well-positioned to develop novel therapeutic strategies. These advancements, combined with a comprehensive approach to patient care, aim to not only regulate disease activity but also enhance the overall quality of life for BU patients. As we move forward, the integration of state-of-the-art research, traditional wisdom, and patient-centered care will pave the way for a brighter future for those battling BU.

## Data Availability

No datasets were generated or analysed during the current study.

## Declarations

### Take-home messages

- Much progress has been made in the study of BU, but there are still gaps in the understanding of its pathogenesis and the optimization of treatment options.
- Recent studies have identified the interaction of genetic, immune, and environmental factors, emphasizing the important influence of microbial elements. In addition, the therapeutic potential of biologics and natural products has provided a new dimension to the treatment paradigm of BU.
- These advances not only fill the gaps in existing research, but also herald the future of subtle and effective BU treatments, emphasizing the value of integrative therapeutic strategies.
